# Onychomadesis: A Rare Manifestation of Coxsackievirus A6 Infection in an Adult Patient

**DOI:** 10.7759/cureus.107987

**Published:** 2026-04-29

**Authors:** Chrystalla Panayiotou, Argyro Marda, George Kyritsis, Stamatina Pagoni, Dimitra Koutete

**Affiliations:** 1 Internal Medicine, General Hospital of Athens "G. Gennimatas", Athens, GRC; 2 Internal Medicine, School of Medicine, National and Kapodistrian University of Athens, Athens, GRC

**Keywords:** adult viral infections, beau’s lines, coxsackievirus a6, hand-foot-and-mouth disease, hfmd, nail matrix arrest, onychomadesis, viral exanthems

## Abstract

Hand, foot, and mouth disease (HFMD) is a self-limiting viral illness predominantly affecting children under 10 years of age, with peak incidence in those aged one to four years. Recent outbreaks of Coxsackievirus A6 (CVA6) have increased its incidence across all age groups, including adults. Onychomadesis, a proximal nail plate detachment due to transient matrix arrest, is a recognized but underreported delayed complication of HFMD. We report a case of a 60-year-old man who developed onychomadesis three weeks after clinical recovery from CVA6-induced HFMD. Initial symptoms included fever, sore throat, gastrointestinal disturbances, and vesiculopapular lesions on the hands, feet, scalp, and oropharynx. He later presented with Beau’s lines, onycholysis, and complete nail shedding involving all 10 fingernails and two toenails on each foot. Conservative management led to complete nail regrowth within eight to 12 weeks. Although the pathophysiology of HFMD-associated onychomadesis is not fully understood, potential mechanisms include direct viral cytotoxicity, localized inflammation, and systemic stress. CVA6 has been increasingly linked to adult cases, often with more severe mucocutaneous and nail manifestations compared to other serotypes. This case highlights CVA6 as an important and emerging cause of onychomadesis in adults following HFMD. Clinician awareness is essential for timely recognition, appropriate patient reassurance, and avoidance of unnecessary investigations. Further studies are needed to explain the underlying mechanisms and risk factors.

## Introduction

Hand, foot, and mouth disease (HFMD) is an acute viral illness that occurs most commonly in young children, with peak incidence in those aged one to four years and the majority of cases arising in children under 10 [1,2]. It is caused by enteroviruses of the Picornaviridae family, with Coxsackievirus A16 (CVA16) and Enterovirus 71 (EV71) being the most frequently responsible pathogens. Various other HEV-A serotypes, as well as certain HEV-B strains, have also been associated with outbreaks [1-3]. Clinically, HFMD is characterized by vesicular eruptions on the hands, feet, and oral mucosa. Although traditionally considered a pediatric infection, HFMD is increasingly recognized in adults, with several recent worldwide outbreaks, particularly those linked to Coxsackievirus A6 (CVA6), highlighting this shift [4,5]. Surveillance data from Europe and Asia over the past decade document a growing proportion of adult cases attributable to CVA6, which is now the predominant serotype in many recent outbreaks [4,5]. Despite the growing number of adult cases, the potential complications of HFMD in this population remain less thoroughly described than in children [6].

Rarely, HFMD can lead to delayed complications affecting the nails, including onychomadesis [7]. Onychomadesis is the proximal separation of the nail plate from the nail matrix, the germinal region at the base of the nail responsible for nail plate production, while remaining attached to the nail bed, sometimes culminating in shedding. Because the nail matrix is a site of rapid cellular proliferation, it is particularly susceptible to transient disruption by systemic metabolic stress or direct viral cytotoxicity associated with acute infections such as HFMD [8,9]. Crucially, this disruption only becomes clinically apparent as a defect in the emerging nail plate weeks after the patient has clinically recovered from the acute illness, making the temporal association easy to overlook [7,10].

Onychomadesis has been associated with a variety of triggers, including infections, autoimmune conditions, critical illness, and certain medications (notably chemotherapeutic agents, anticonvulsants, and retinoids) [8]. Among infectious causes, HFMD is one of the most frequently reported [7,8]. While HFMD-associated onychomadesis was historically linked predominantly to CVA16 in pediatric populations, more recent outbreaks show that CVA6 is now the predominant serotype associated with this complication in both children and adults [1,4,5]. CVA6 is characterized by a broader and more atypical mucocutaneous distribution compared to CVA16 and EV71, frequently involving the scalp, buttocks, and perioral region in addition to the classic acral and oral sites [4-6]. This wider tissue tropism may underlie CVA6’s propensity for more severe nail involvement. Awareness of these associations is important for recognition, prognosis, and management, particularly when onychomadesis arises as a delayed complication of viral infections such as HFMD [10].

## Case presentation

A 60-year-old male primary school teacher presented with a three-day history of acute illness. His occupation entailed regular, close contact with young children in a classroom setting, representing a plausible epidemiological source of enteroviral exposure, although no specific case of HFMD was identified among his students at the time. His medical history included dyslipidemia, nephrolithiasis, benign prostatic hyperplasia (BPH), and left bundle branch block (LBBB). He had also undergone two prior surgical excisions of squamous cell carcinoma of the scalp. His regular medications were rosuvastatin 10 mg for dyslipidemia and alfuzosin 10 mg for lower urinary tract symptoms related to his BPH. Neither medication has a recognized association with nail matrix arrest or onychomadesis.

The patient presented with a fever up to 38.0 °C, sore throat, and gastrointestinal disturbances comprising nausea, vomiting, and diarrhea, all of which resolved within the first week. These symptoms were accompanied by a widespread rash consisting of maculopapular and vesicular lesions on the hands, feet, palms, soles, and scalp, a distribution consistent with the atypical mucocutaneous involvement characteristic of CVA6, as well as enanthem with vesicles and ulcerative lesions in the oropharynx.

Laboratory investigations on presentation are summarized in Table 1. The white cell count was within normal limits at 6.47 × 10³/μL, with relative lymphopenia (16.17%) and mild monocytosis (11.74%), consistent with an acute viral syndrome. Inflammatory markers were markedly elevated: CRP 117.2 mg/L (ref <5), erythrocyte sedimentation rate (ESR) 57 mm/h (ref 0-30), and fibrinogen 646 mg/dL (ref 200-400), reflecting a significant systemic inflammatory response. Creatine phosphokinase (CPK) was mildly elevated at 342 IU/L (ref 30-200), likely attributable to the acute viral illness; renal and hepatic function, electrolytes, and glucose were all within normal limits. A SARS-CoV-2 antigen test was negative. PCR testing performed on both serum and a pharyngeal swab identified CVA6 as the causative pathogen. Testing for herpes simplex virus (HSV) 1/2 and varicella-zoster virus (VZV) by PCR was negative, excluding alternative vesicular viral etiologies. The patient recovered clinically from HFMD within one week.

**Table 1:** Selected laboratory investigations on presentation Virological testing confirmed acute CVA6 infection; HSV, VZV PCR, and SARS-CoV-2 antigen were negative. Elevated inflammatory markers and mild CPK elevation are consistent with an acute viral syndrome. CPK, creatine phosphokinase; CVA6, Coxsackievirus A6; ESR, erythrocyte sedimentation rate; HSV, herpes simplex virus; VZV, varicella-zoster virus

Laboratory parameter	Result	Units	Reference range
Virological			
CVA6 PCR (serum and pharyngeal swab)	Positive	-	Negative
HSV 1/2 PCR	Negative	-	Negative
VZV PCR	Negative	-	Negative
SARS-CoV-2 antigen (nasopharyngeal swab)	Negative	-	Negative
Hematology and inflammatory markers			
White blood cell count	6.47	×10³/μL	4.00-10.00
Lymphocytes	16.17	%	20.50-51.10
Monocytes	11.74	%	1.70-9.30
ESR	57	mm/h	0-30
C-reactive protein	117.2	mg/L	<5.0
Fibrinogen	646	mg/dL	200-400
CPK	342	IU/L	30-200

**Figure 1:**
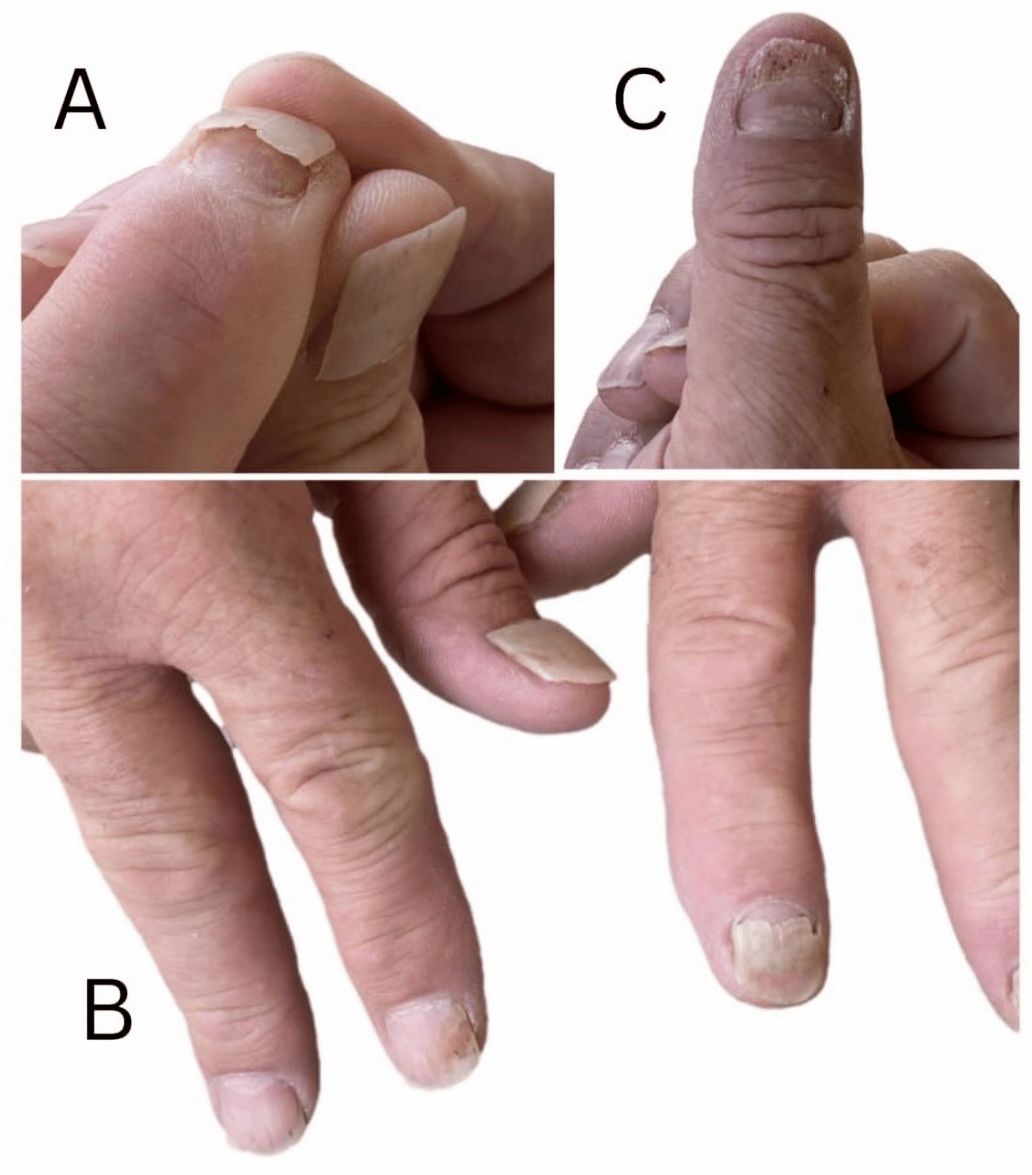
Clinical manifestations of post-HFMD-associated onychomadesis at three weeks post-recovery (A) Close-up view of a fingernail with lifting and partial separation of the nail plate, consistent with onychomadesis. (B) Multiple fingernails showing Beau’s lines, onycholysis, and partial or complete nail shedding. (C) Proximal nail plate detachment and dystrophy of the right thumb. HFMD, hand, foot, and mouth disease

Three weeks after clinical recovery, he returned with complaints of nail abnormalities affecting both hands and feet (Table 2). Physical examination revealed Beau’s lines (transverse grooves indicating the precise timing of matrix arrest), depressions, and partial detachment of the nail plates. All 10 fingernails were affected, along with two toenails on each foot (four toenails in total). In several nails, complete shedding had occurred (Figure 1). These findings were consistent with onychomadesis as a delayed complication of HFMD [11].

**Table 2:** Timeline of clinical events in a 60-year-old male with CVA6-associated HFMD and subsequent onychomadesis CVA6, Coxsackievirus A6; HFMD, hand, foot, and mouth disease

Timepoint	Clinical event
Days 1-3	Onset of fever (≤38.0 °C), sore throat, nausea, vomiting, diarrhea, and widespread vesiculopapular rash (hands, feet, palms, soles, scalp, and oropharynx)
Day 7	Complete clinical recovery from acute HFMD illness
Week 4 (three weeks post-recovery)	Onset of nail changes: Beau’s lines (transverse grooves), onycholysis, and partial or complete nail shedding affecting all 10 fingernails and two toenails on each foot (four toenails in total)
Weeks 8-12	Progressive and complete nail regrowth with restoration of normal nail architecture; no residual dystrophic changes

Alternative causes of onychomadesis were systematically considered and excluded. There was no history of nail trauma, systemic illness other than the preceding HFMD episode, or use of medications known to cause nail matrix arrest. There were no clinical or serological features of autoimmune disease and no history or examination findings suggestive of pemphigus vulgaris. The clear temporal relationship between the PCR-confirmed CVA6 infection and the subsequent nail changes strongly supported a postinfectious etiology.

Notably, the patient had not had HFMD lesions directly adjacent to all of the affected nails during the acute episode, underscoring that periungual involvement during the acute phase is not required for onychomadesis to develop, a finding consistent with the literature [12,13].

**Figure 2:**
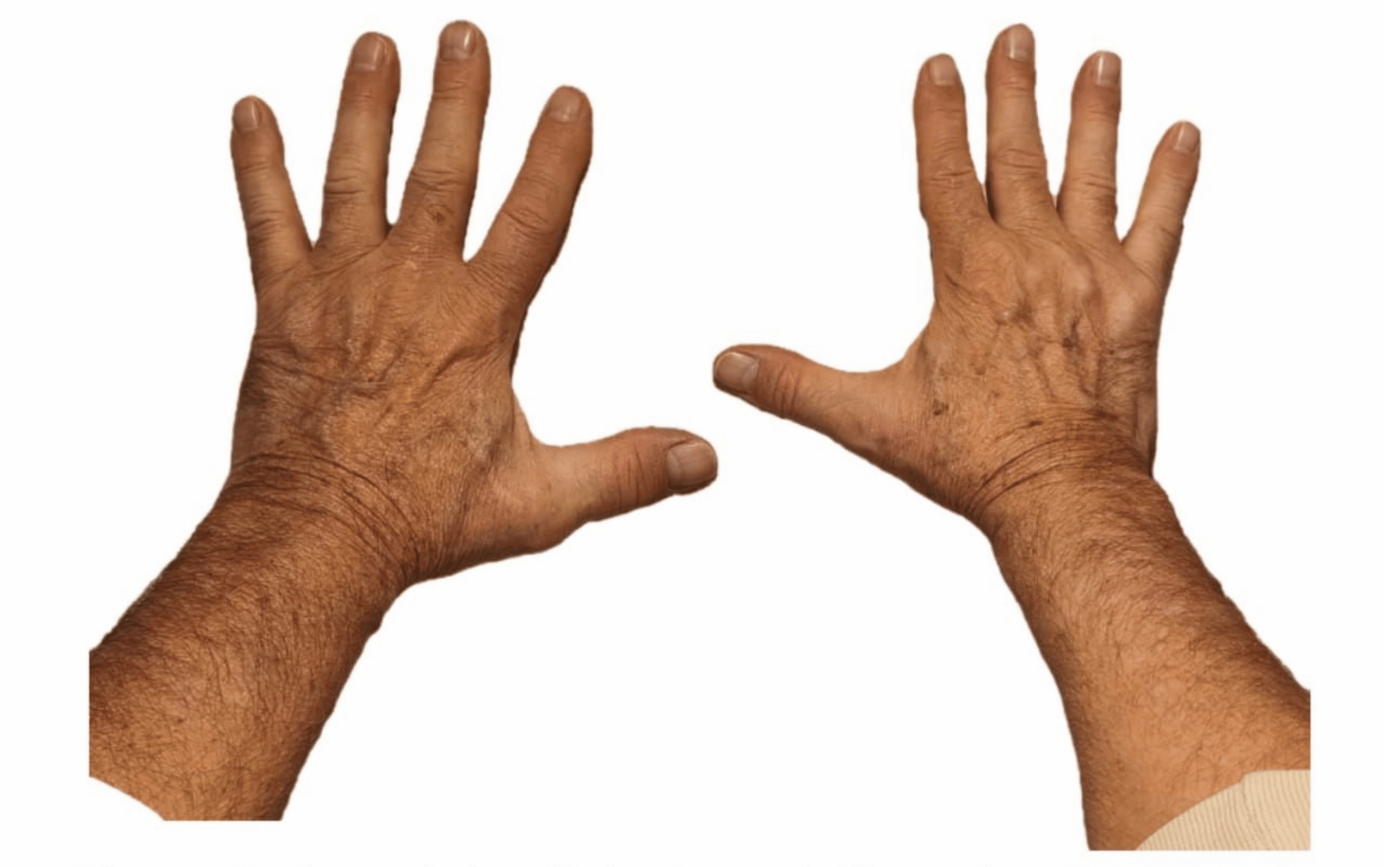
Complete clinical resolution of nail abnormalities following postinfectious onychomadesis at 12 weeks post-recovery Photograph of both hands obtained 12 weeks after HFMD-associated onychomadesis, demonstrating full regrowth of the nail plates with restoration of normal nail architecture and no residual dystrophic changes. HFMD, hand, foot, and mouth disease

The patient was managed conservatively with reassurance, protection of the affected nails, and avoidance of trauma. Follow-up over the subsequent eight to 12 weeks demonstrated gradual and complete regrowth of all affected nails, with restoration of normal nail architecture and no further complications (Figure 2).

## Discussion

Onychomadesis is characterized by proximal separation of the nail plate resulting from a temporary arrest of nail matrix activity [9]. Although multiple etiologies have been described, including infections, autoimmune disorders, critical illness, and certain medications, HFMD has emerged as one of the most common infectious triggers [7,8]. While historically HFMD-associated onychomadesis was reported mainly in pediatric populations (often linked to CVA16), more recent outbreaks demonstrate that CVA6 is now the predominant serotype associated with post-HFMD onychomadesis in both children and adults [1,4,5]. CVA6-associated cases frequently present with more widespread or atypical mucocutaneous involvement and a more striking nail phenotype compared to classic HFMD caused by CVA16 or EV71 [6]. In this context, our case adds meaningful clinical documentation by illustrating the characteristic nail changes following confirmed CVA6 infection in an adult patient; to date, reports of HFMD-associated onychomadesis in adults have been largely limited to isolated case studies, with CVA6 identified in many [3,14].

Although several mechanisms have been proposed, including direct viral cytotoxicity at the nail matrix, inflammation-mediated suppression of matrix keratinocyte activity (e.g., from periungual vesicular lesions), and systemic stress responses (such as high fever), the pathophysiology of onychomadesis remains incompletely understood [15]. The typical delay of three to eight weeks between acute infection and the onset of nail changes aligns with the expected timeline for matrix injury to manifest distally, yet the precise biological pathways responsible for matrix arrest have not been fully elucidated. Notably, onychomadesis has been observed even in the absence of significant fever or periungual HFMD lesions, indicating that a simple “systemic shock” or local trauma explanation is insufficient [12,13]. Conversely, some reports document onychomadesis developing only in nails that had adjacent HFMD eruptions, supporting a role for localized inflammation [12]. In one study, CVA6 RNA was detected by RT-PCR in shed nail fragments, suggesting that direct viral replication in the nail matrix may contribute to matrix arrest [15]. Overall, the variability in clinical severity and nail involvement underscores a multifactorial process [8,10].

In our patient, the clear temporal relationship between the acute HFMD episode and the subsequent development of Beau’s lines, onycholysis, and complete nail shedding strongly supports a diagnosis of postinfectious onychomadesis [11]. His underlying comorbidities, such as dyslipidemia, nephrolithiasis, BPH, LBBB, and prior excisions of squamous cell carcinoma, have no known associations with nail matrix arrest. His regular medications, rosuvastatin and alfuzosin, similarly have no recognized association with onychomadesis, further supporting the postinfectious etiology. The mildly elevated CPK and significantly raised acute-phase reactants (CRP 117.2 mg/L, fibrinogen 646 mg/dL, and ESR 57 mm/h) are consistent with the systemic inflammatory response to acute CVA6 infection and do not suggest an alternative diagnosis. The active exclusion of autoimmune, traumatic, and other infectious causes, combined with PCR-confirmed CVA6 infection, establishes a robust clinico-etiological link. The clinical course aligns with previous reports describing spontaneous resolution over several months as new nail growth progresses [2,9].

Given the growing number of global reports implicating CVA6 in adult HFMD-associated onychomadesis [4,5], this case reinforces the importance of clinician awareness. Importantly, the diagnosis should not be excluded solely on the basis of absent periungual lesions during the acute episode. Further research is warranted to clarify the mechanisms, risk factors, and virological or host determinants that predispose certain individuals to nail matrix arrest following HFMD [3,6,14,15].

## Conclusions

This case demonstrates that CVA6 can lead to onychomadesis as a delayed complication of HFMD in adults, an association that is increasingly recognized yet may still be overlooked in clinical practice. Although the nail changes may appear alarming, the condition is generally benign and self-limiting, with complete nail regrowth expected over eight to 12 weeks with conservative management alone. Recognizing this link is crucial to avoid unnecessary investigations and to provide appropriate reassurance regarding the expected full recovery of the nails.

From a broader clinical and public health perspective, heightened awareness is particularly warranted in settings experiencing CVA6 outbreaks, both in pediatric and adult populations. Primary care providers and emergency physicians should be familiar with this association, as patients may present to nonspecialist settings weeks after the acute illness has resolved. A brief history of a febrile vesicular exanthem in the preceding four to eight weeks in a patient presenting with unexplained nail shedding should prompt consideration of post-HFMD onychomadesis. In cases where the clinical picture is atypical or uncertain, early involvement of dermatology and/or infectious disease specialists may be beneficial. Importantly, because the pathophysiological mechanisms underlying post-HFMD onychomadesis remain incompletely understood, further research is needed to clarify why matrix arrest occurs in only a subset of patients and whether viral, host-related, or immunological factors contribute.
